# Aseptic meningitis following a bupivacaine spinal anesthesia

**DOI:** 10.11604/pamj.2017.27.192.9327

**Published:** 2017-07-13

**Authors:** Nawfal Doghmi, Amine Meskine, Aziz Benakroute, Mustapha Bensghir, Abdelouahed Baite, Charki Haimeur

**Affiliations:** 1 Service d’Anesthésie-Réanimation, Hôpital Militaire Med V, Rabat, Maroc

**Keywords:** Aseptic meningitis, bupivacaine, spinal anesthesia

## Abstract

Spinal anesthesia complicated by meningitis is rare. The diagnosis is difficult and the clinical signs are unspecific. There is a subgroup called aseptic meningitis of a different mechanism (hypersensitive reaction and irritation of the meninges), which must be identified for appropriate care. We report the case of aseptic meningitis resulting from bupivacaine use complicating spinal anesthesia. She is 31 years old and was admitted to the intensive care unit for meningitis following a Caesarean delivery. 10 hours after the procedure, she was found to have severe headache, neck stiffness and was found restless. She lost consciousness; she was treated by attending physicians. A CT scan have been performed and was found normal. 24 hours after intubation, the patient woke up. The clinical and biological valuations were normal, allowing for the elimination of the other causes of meningitis.

## Introduction

Spinal anesthesia is a technique which improves a lot the survey and the quality of post-operative care in surgery, especially in obstetrics. Complications due to this technique are few, but when they occur, they are very serious. One of these complications is meningitis or meningoencephalitis, the diagnostic of such complications is difficult to make due to changes caused by surgery. Causes of meningitis can commonly be classified to two kinds: infectious and non-infectious, but another kind can occur especially due to spinal or epidural anesthesia, it is chemical or aseptic meningitis [1, 2]. The diagnosis of aseptic meningitis is difficult to make, it is an elimination diagnosis, and the management of chemical meningitis is different than others. We report here a case of meningoencephalitis occurred following a bupivacaine spinal anesthesia for Caesarean section. We discuss the strategy of diagnosis and the management of aseptic meningitis.

## Patient and observation

**Figure 1:**
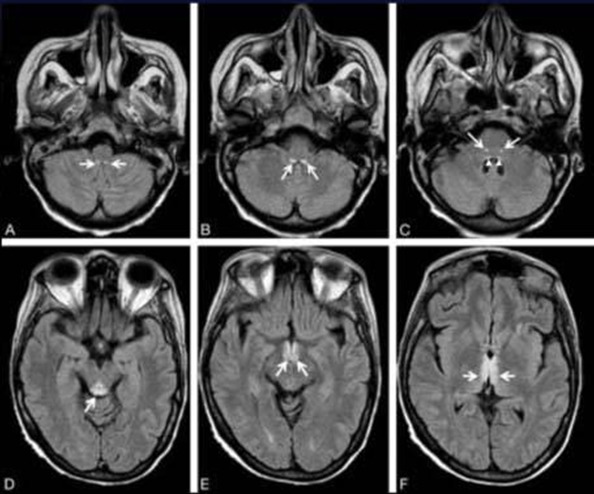
MRI of the head showing a diffuse hyper intensity of leptomeningeal spaces

A 31 year old, G3P2 at 39 week gestational age presented for prophylactic caesarean. Her past medical history was unremarkable; at the time of admission, she denied any recent history of upper respiratory or urinary tract infection; antibiotics treatment or vaccination. Her pre-operative evaluation concludes of an ASA I patient. Following hydration with 500ml of saline solution, the patient was placed (sitting position); the anesthetist wore a cap, mask, and sterile gloves for the procedure. The lumbar area was prepared using povidone iodine 10% spray contained in a spray bottle and draped in a sterile fashion. The spinal anesthesia was performed in L3-L4 interspace, an 90mm 25-gauge Whitecare spinal needle was passed through the Tuohy needle and after a free flow of clear CSF, a mixture of 2,5ml (12,5mg) of bupivacaine 0,5% and 0,5ml (25µg) of fentanyl was injected into the subarachnoid space. The patient had also received 2g of Cefazoline. The peroperative period was stable; the patient did not present any hemodynamic or respiratory failure. The patient gave birth to a healthy male baby weighing 3200g with a one minute Apgar score of 10, the operation was carried out without incident and the patient return to her hospital room 3hours after the end of the procedure. The post-operative analgesia was performed by the association of paracetamol, nefopam and ketoprofen. 10 hours after the spinal anesthesia, the patient had a severe headache, agitation and loss of consciousness. The clinical exam found an apyrexia with a temperature of 36,7°c , a blood pressure of 125/65mmHg , a rigidity of the neck, a GCS of 8/15 and no other focal neurological deficits were noted; the patient was intubated and transferred to the ICU, she was sedated by fentanyl and midazolam. We were concerned that she had suffered a subarachnoid haemorrhage, a stroke or meningitis. A full blood count showed a haemoglobin concentration at 12g/dl, WBC 10500/mm and platelets 175.000/mm. A normal glucose level at blood was also found (1,15g/dl). A CT scan was performed which showed no abnormality, an MRI was also performed and showed a diffuse hyper intensity of leptomeningeal spaces (Figure 1). The EEG showed a diffuse electrical disorder of the brain and no localizing signs. Lumbar puncture revealed a cloudy fluid , with normal pressure, laboratory analysis showed a protein concentration of 4g/L, a normal glucose concentration (0,90g/dl) and WBCs of 2700/mm^3^ with 95% PNN, 5% lymphocytes, the search of soluble antigen was negative. An empirical antibiotics were started with ceftraixone 2g every 8h and vancomycine 1g every 12H IV, combined to dexamethasone 10mg/8h IV. The evolution was marked, 24 hours after, by the awakening of the patient after stopping sedation, the clinical exam found apyrexia and no rigidity of the neck and a GCS of 15/15, the patient was extubated. Results of bacterial cultures were negative 48H after the lumbar puncture, even the research of Koch bacillus in spittle was negative, and polymerase chain reaction was performed and found to be negative. Thus, a diagnosis of aseptic meningitis has been made and antibiotics were stopped 48h after lumbar puncture. The patient made an uneventful complete recovery and was discharged from the ICU 3 days after her admission, and she was well at follow up 15 days later, an MRI had also been performed and showed no abnormality.

## Discussion

Meningitis following regional anesthesia is rare, both bacterial and aseptic ones have been reported. The difference between the 2 types is not always easy to make, due to antibiotics taken before the procedure. Aseptic or chemical meningitis has been reported since 1940 sporadically, it is a clinical syndrome characterized by fever or apyrexia, headache, neck stiffness and photophobia. When associated with spinal anesthesia, it has an acute onset within 24H of Dural puncture and a self-limiting course [3, 4]. Harding et al, reported a case of aseptic meningitis after spinal anesthesia in obstetrics, and attributed that to a contaminant, usually a detergent used in sterilizing the spinal needles and syringes [3–7]. Lambott and al, in his case, attribute the cause to hypersensitivity reaction or direct irritation of the meninges by a product injected to their contact. He also reported in his case, that the responsible product is bupivacaine [8, 9]. In our case, we also have suspected the bupivacaine as the responsible cause of the meningitis. Aseptic meningitis is defined bacteriologically by the absence of any germ at the bacterial culture of the CSF. This definition, is not enough for diagnosis of aseptic meningitis, several elements has to be searched to the purpose diagnosis of it. Lambott and al, in his case, report that the most important element is glucose in CSF, which has to be at a normal value; in contrary of the case of bacterial meningitis which the glucose value in CSF is down. Ducornet and al [10], also defined a several conditions to a diagnosis of aspetic meningitis: time to onset of signs within 24 hours after spinal anesthesia; the absence of prior antibiotic therapy; apyrexia; a WBCs in CSF similar to bacterial meningitis; high protein value in CSF; a normal glucose value in CSF; absence of identified germ in bacterial culture; full recovery less than 48hours. In our case, the diagnosis of aseptic meningitis was due to absence of germ, the apyrexia, the onset of signs was 10 hours after spinal anesthesia, the normal glucose value and the recovery has been in less than 48hours with the arrest of antibiotics. However, the diagnosis of aseptic meningitis stays a diagnosis of exclusion, even when the clinical picture suggests aseptic meningitis, it is imperative that antibiotics are given to prevent the possible disastrous consequences of untreated bacterial meningitis. This case illustrates the importance of having a broad differential in patients with meningeal signs after spinal anesthesia.

## Conclusion

Aseptic meningitis is a rare and uncommon complication of spinal anesthesia; its diagnosis is an exclusion one. However, if there is any doubt postoperatively in a patient with these complaints, it is imperative to performa timely examination of the CSF to initiate prompt medical treatment and prevent further complications.

## Competing interests

The authors have no competing interests to declare.
